# Biogenic Silver Nanoparticles Decorated with Methylene Blue Potentiated the Photodynamic Inactivation of *Pseudomonas aeruginosa* and *Staphylococcus aureus*

**DOI:** 10.3390/pharmaceutics12080709

**Published:** 2020-07-29

**Authors:** Paramanantham Parasuraman, Thamanna R. Y, Chitra Shaji, Alok Sharan, Ali H. Bahkali, Helal F. Al-Harthi, Asad Syed, V.T. Anju, Madhu Dyavaiah, Busi Siddhardha

**Affiliations:** 1 Department of Microbiology, School of Life Sciences, Pondicherry University, Puducherry 605014, India; parasupondiuni@gmail.com (P.P.); thamannayousuf@gmail.com (T.R.Y); 2 Department of Physics, School of Physical, Chemical & Applied Sciences, Pondicherry University, Puducherry 605014, India; chitrashaji@gmail.com (C.S.); alok.phy@pondiuni.edu.in (A.S.); 3 Department of Botany and Microbiology, College of Science, King Saud University, P.O.Box 2455, Riyadh 11451, Saudi Arabia; abahkali@ksu.edu.sa (A.H.B.); aboragad-797@hotmail.com (H.F.A.-H.); 4 Department of Biochemistry and Molecular Biology, School of Life Sciences, Pondicherry University, Puducherry 605014, India; ansh4378@gmail.com (V.T.A.); madhud14@yahoo.co.in (M.D.)

**Keywords:** multidrug resistance, antimicrobial photodynamic inactivation, silver nanoparticles, methylene blue, anti-biofilm

## Abstract

The persistence of multidrug resistance among microorganisms has directed a mandate towards a hunt for the development of alternative therapeutic modalities. In this context, antimicrobial photodynamic therapy (aPDT) is sprouted as a novel strategy to mitigate biofilms and planktonic cells of pathogens. Nanoparticles (NPs) are reported with unique intrinsic and antimicrobial properties. Therefore, silver NPs (AgNPs) were investigated in this study to determine their ability to potentiate the aPDT of photosensitizer against *Staphylococcus aureus* and *Pseudomonas aeruginosa*. Biologically synthesized AgNPs were surface coated with methylene blue (MB) and studied for their aPDT against planktonic cells and biofilms of bacteria. The nano-conjugates (MB-AgNPs) were characterized for their size, shape and coated materials. MB-AgNPs showed significant phototoxicity against both forms of test bacteria and no toxicity was observed in the dark. Moreover, activity of MB-AgNPs was comparatively higher than that of the free MB, which concludes that MB-AgNPs could be an excellent alternative to combat antibiotic resistant bacteria.

## 1. Introduction

The emergence of several new diseases and multidrug resistant (MDR) bacterial strains has accelerated the drug discovery program for the search of novel drugs. Advancements in science and technology have facilitated the development of early diagnosis, better prevention, and advanced treatment options to control the disease [1]. Antibiotics are known as one of the major therapeutic agents employed in the treatment of infectious diseases. However, development of antibiotic resistance by infectious microorganisms leads to the failure of these therapeutic agents [2]. Center for Disease Control and Prevention reported that the world is standing on the edge of a post-antibiotic era where more deaths are due to bacterial infections than cancer. The acquired resistance towards more than one antibiotic is a key challenge in MDR strains elimination program. The ESKAPE group of pathogens including *Enterococcus faecium, Staphylococcus aureus, Klebsiella pneumoniae, Acinetobacter baumannii, Pseudomonas aeruginosa*, and *Enterobacter* spp. are major causes of bacterial and nosocomial infections and often “escape” from antimicrobial treatment by developing resistance mechanisms against current antimicrobial agents [3]. This is a serious concern associated with the increasing global rate of mortality and morbidity [4]. In the pre-penicillin era, a substantial patient mortality rate was observed because of uncontrollable microbial infections. Recently, new generation antibiotics failed to control microbial infections owing to their multidrug resistance [5]. Hence, alternative therapeutic approaches for the treatment of multidrug resistant bacteria are highly required. Recently, several approaches have been introduced in medicine to develop effective treatment practices against drug-resistant bacteria. Two prominent approaches are the application of metallic nanoparticles (NPs) and antimicrobial photodynamic therapy (aPDT), both of which are considered to be reliable alternatives to antimicrobial chemotherapy. One of the metallic nanoparticles, silver NPs (AgNPs) are widely accepted as potential antimicrobial agents that effectively disrupt the bacterial cell wall and alter intracellular balance [6,7]. There are some limitations associated with AgNPs employed as antimicrobial agents such as expensive synthesis procedures and generation of toxic byproducts through physical and chemical synthetic routes. To overcome these limitations, biological routes have gained more importance for the synthesis of AgNPs. Biological entities used for the synthesis of AgNPs are bacteria, fungi, and plant extracts. Among these, bacteria-mediated synthesis of AgNPs is considered a beneficial biological route of synthesis owing to the simple synthesis process, lower production costs, and non-toxic nature [8].

Another alternative strategy that can be employed to eradicate biofilms is light-based therapy called aPDT. This involves the internalization of photosensitizers (PS) by bacteria which gets photo activated with a light of suitable wavelength. Upon illumination with light, PS get excited to a triplet state that interacts with oxygen existing in and around the cells to generate cytotoxic reactive oxygen species (ROS). ROS is produced through type I or type II photochemical mechanisms. Type I and type II pathways generate hydroxyl radials and singlet oxygen through electron and energy transfer mechanisms, respectively [9]. Reactive oxygen species generated through aPDT cause oxidative death of microorganisms. ROS can oxidize the cell membrane that alters the membrane integrity and oxidize biomolecules (DNA, proteins, enzymes, and lipids). Recently, aPDT has become widely accepted as a therapeutic modality for chronic infections. The prominent advantage of repetitive aPDT treatment over traditional antibiotic therapy is the elimination of resistance strains. This is because the free radicals generated through aPDT preclude the selection of antibiotic strains by interacting with different metabolic pathways of target bacteria [10]. Moreover, aPDT has shown significant effects on infectious bacteria that are difficult to treat by conventional antibiotics. One of the phenothiazinium dye widely employed for aPDT is methylene blue (MB). This dye is one of the photosensitizers studied for broad spectrum antibacterial activity. MB is a globally approved dye and is extensively used in clinical practices. Moreover, activated MB produces a higher quantum yield of singlet oxygen (Φ∆ of approximately 0.5). MB is comparatively less toxic, and the action of dye varies according of the type of microorganisms. The negatively charged bacterial membrane interacts with a positively charged PS, generating a electrostatic interaction and eventually resulting in cellular damage [11]. MB induces phototoxicity through the type I or type II pathway [12]. Scientists have reported the effectiveness of MB in aPDT against several bacteria such as methicillin-resistant *S. aureus*, *S. aureus*, vancomycin-resistant *Enterococci*, *S. epidermidis*, *Streptococcus pyogenes*, *Propionibacterium acnes*, *Corynebacterium minutissimum*, and *Escherichia coli.* Furthermore, it has also been employed in the treatment of skin, nail, and mucous membrane infections caused by *Candida albicans* [13].

Although MB is widely accepted as a promising PS, application of this dye in aPDT is limited due to several reasons such as poor penetration, poor bioavailability, and short retention time against the target microorganism [8,9]. Moreover, establishment of bacterial colonies in biofilms renders the PS less permeable because of the presence of extracellular polymeric matrix, which limits the permeability and diffusion of PS. Conjugation of MB onto metallic NPs addresses these limitations. In order to enhance the effectiveness of aPDT, nanoparticles are employed as dye carriers. Nanoparticles encapsulated with photosensitizers of opposite charge helps optimize aPDT regimes for better results. Nano-dye conjugates exhibit a higher lethality than their free counterparts due to the synergistic activity of nanoparticles and photodynamic therapy. In this context, silver nanoparticles are previously employed to conjugate PS to enhance the aPDT due to the synergistic action of silver and PS [14]. Silver nanoparticles are one of the widely studied metallic nanoparticles as PS carriers in aPDT [15]. In a study, toluidine blue O (another phenothiazinium dye) conjugated silver nanoparticles inhibited the biofilm formation and biofilm associated gene expression in *Streptococcus mutans* compared to the free dye alone [14]. In a recent study, MB conjugated on gold nanoparticles exhibited phototoxicity on MDR strains through the type II pathway. Nanoparticles conjugated with MB exhibited 97% of bactericidal action on MDR clinical strains due to the enhanced pharmacokinetic and pharmacodynamic properties of dye after conjugation [12]. In previous reports, a composite of MB and gold NPs enhanced the delivery of PS into the cell and enhanced the antimicrobial photodynamic inactivation [16]. In another report, the photosensitizer zinc phthalocyanine was integrated with TiO_2_ nanoparticles and investigated for its use as antimicrobial photodynamic therapy against *S. aureus.* The nanoconjugate showed promising phototoxicity towards *S. aureus* [17]. Based on a literature survey, MB and metallic NPs can be employed in combination to eliminate microorganisms using light irradiation [18,19]. Considering the above findings, biologically synthesized AgNPs were surface decorated with MB and evaluated for their efficacy by aPDT against *P. aeruginosa* PAO1 and *S. aureus*. In recent years, several researches have focused on the development of therapeutic alternatives and their mode of actions against MDR strains but very few have targeted ESKAPE specifically. This study explains the alternative therapeutic method against *P. aeruginosa* PAO1 and *S. aureus,* major pathogens from an ESKAPE group of pathogens.

## 2. Materials and Methods

### 2.1. Chemicals

All chemicals and bacterial culture media (Luria Bertani broth, LB) were obtained from HiMedia Laboratories Pvt. Ltd., Mumbai, India; Alfa Aesar, Mumbai, India; and Sigma-Aldrich, Bengaluru, India.

### 2.2. Test Bacteria and Growth Conditions

Bacterial isolate *B. subtilis* RY3 was used to synthesize the biogenic silver nanoparticles. The test culture *S. aureus* (MCC 2408) was procured from Microbial Culture Collection (MCC), National Center for Cell Science, Pune, India. Another test bacterium, *P. aeruginosa* PAO1 was a kind gift from Professor E. Peter Greenberg (University of Washington), Seattle, WA, USA.

### 2.3. Isolation and Identification of Bacterial Isolates

Sewage water samples collected from Kalapet, Puducherry, India were used to isolate bacteria. The samples were subjected to dilution in 0.9% normal saline and seeded onto nutrient agar plates. The nutrient agar plates were kept in an incubator for 48 h at 37 °C. The obtained colonies grown on culture plates were further screened for their ability to synthesize AgNPs. The bacterial isolates were screened by adding 1 mM AgNO_3_ solution to the culture supernatant, incubated under sunlight for 30 min, and observed for visible color change [20].

The 16s rDNA sequencing-based method was employed to identify the bacterial isolate named RY3 which showed promising color change in the screening process. The amplification of 16s rDNA of the bacterial isolate was performed using the primers 518F (5′ CCAGCAGCCGCGGTAATACG 3′) and 800R (5′ TACCAGGGTATCTAATCC 3′). The obtained product was sequenced at Macrogen Inc., Seoul, South Korea. BLAST analysis of the sequence was performed in NCBI database. Form the NCBI database, sequences highly similar to the bacterial isolate RY3 were selected to construct the phylogenetic tree [21]. The neighbor-joining method was adopted to construct the phylogenetic tree using MEGA 5.0 software [22].

### 2.4. Extracellular Synthesis of AgNPs

The bacterial isolate RY3 was grown in nutrient broth with culture conditions of 37 °C, 48 h and 100 rpm as shaking conditions. Bacterial cells were removed after centrifugation for 10 min at a centrifugal speed of 10,000 rpm. The culture supernatant obtained after centrifugation was used for extracellular synthesis of AgNPs, wherein 1 mM AgNO_3_ solution was mixed with the culture broth (*v*/*v*). The resulted mixture was incubated at a shaking condition of 150 rpm for 24 h at 37 °C. A control experiment was executed in parallel with sterile nutrient broth and AgNO_3_ solution separately. The experimental setups were exposed to bright light and observed for visible color change in the reaction mixture. The reaction mixture was observed periodically to examine color change [20]. The synthesized nanoparticles were thoroughly cleaned with sterile water and dried and stored in an airtight container at room temperature.

### 2.5. Characterization of AgNPs

#### 2.5.1. UV-Visible (UV-Vis) Spectroscopy

The bio-reduction of AgNO_3_ in the reaction mixture was monitored at different time intervals. UV-Visible spectra were documented in a double beam spectrophotometer (Model: Cary 60, Agilent Technologies India Pvt. Ltd., Chennai, India) at 200 nm to 800 nm with a resolution of 1 nm [23].

#### 2.5.2. Particle Size and Transmission Electron Microscopic (TEM) Analysis

Size distribution of biologically synthesized AgNPs was evaluated using dynamic light scattering (DLS) measurement by dissolving it in water [24]. The biologically synthesized AgNPs were further subjected to transmission electron microscopic analysis. The nanoparticle solution was positioned as a drop on the copper grid coated with carbon and air dried in a vacuum desiccator. TEM micrographs were captured in a JEOL/JEM 2100 instrument (JEOL Ltd. Tokyo, Japan), working in a 200 kV of acceleration voltage [25]. The dimension and morphology of the synthesized AgNPs were analyzed from the obtained TEM micrographs.

#### 2.5.3. Fourier Transform Infrared Spectroscopic Analysis (FTIR)

The synthesized nanoparticles were dried and mixed with potassium bromide at a ratio of 1:100 to analyze the functional groups. The spectrum was documented in a wavenumber range and resolution of 400 to 4000 cm^−1^ and 4 cm^−1^, respectively [21].

#### 2.5.4. X-ray Powder Diffraction (XRD) and SEM-EDX Analysis

The AgNPs powder was carried out through high resolution X-Ray diffraction techniques with Cu Kα radiation (λ = 1.54060 Å) and functioned at 40 Kv and 30 mA at 2θ scan range at 30°–90°. Phase determination was done using standard ICDD (International Centre for Diffraction Data) and card no: 00-004-0783 [26]. Scanning Electron Microscopy (SEM) with Energy Dispersive X-Ray Analysis (SEM-EDX) was performed to identify the presence of Ag in the test sample [27].

### 2.6. Synthesis of MB-AgNPs

MB-AgNPs were prepared by mixing the biologically synthesized AgNPs (1.5 mg/mL) with 20 μg/mL of MB in distilled water. The obtained solution was retained at room temperature for 24 h in continuous shaking conditions. The sample was centrifuged for 40 min at 6000 rpm to obtain the pellet. The obtained pellet was washed three times and dissolved in water for further characterization [19].

### 2.7. Characterization of MB-AgNPs

A double-beam UV-Visible spectrophotometer (Cary 60, Agilent Technology) was used to characterize the MB-AgNPs. Absorbance was recorded at a wavelength range of 300–800 nm. Functional group analysis of MB, AgNPs, and MB-AgNPs was carried out in the FTIR instrument with a resolution and wavenumber range of 4 cm^−1^ and 400–4000 cm^−1^, respectively [19].

### 2.8. In Vitro Release of MB

The release of MB from nano dye conjugates was measured in distilled water at neutral pH (7.0). MB-AgNPs suspension (500 µg/mL) was prepared and incubated (with a temperature of 37 °C) in an orbital shaker (125 rpm). At fixed intervals (30 min), aliquots of 1 mL test suspensions were collected and centrifuged (12,000 rpm for 20 min). The optical density of dye released into supernatant was documented at 668 nm. Dye release was calculated using the standard curve of MB [28].

(1) $$\mathrm{Amount}\;\mathrm{of}\;\mathrm{dye}\;\mathrm{released}\;\left(\%\right)=\frac{\mathrm{Amount}\;\mathrm{of}\;\mathrm{dye}\;\mathrm{in}\;\mathrm{the}\;\mathrm{supernatant}}{\mathrm{Amount}\;\mathrm{of}\;\mathrm{dye}\;\mathrm{loaded}\;\mathrm{onto}\;\mathrm{AgNPs}}\times 100$$

### 2.9. Methylene Blue Uptake

The amount of dye that was taken up by both bacteria was investigated through this study. About 100 µg/mL of free MB and MB-AgNPs were added to the bacterial culture (0.5 McFarland standard) and kept at room temperature in the dark for 2 h with continuous shaking (125 rpm). The bacterial cell pellets were collected after centrifugation (1000 rpm, 10 min) and washed gently with phosphate buffer saline (PBS). MB bound to the bacteria was extracted with methanol (10,000 rpm for 20 min). The amount of MB present in the cell extract was calculated spectrophotometrically at 668 nm using the standard curve of free MB dissolved in distilled water [28]. Bacterial uptake of MB was determined using the following equation:

(2) $$\mathrm{MB}\;\mathrm{uptake}\;\left(\%\right)=\frac{\mathrm{Quantity}\;\mathrm{of}\;\mathrm{MB}\;\mathrm{present}\;\mathrm{in}\;\mathrm{the}\;\mathrm{pellet}}{\;\mathrm{Total}\;\mathrm{quantity}\;\mathrm{of}\;\mathrm{MB}\;\mathrm{used}\;\mathrm{for}\;\mathrm{experiment}}\times 100$$

### 2.10. Light Source

A diode laser of 660 nm wavelength light with an output power of 132 mW was used as a light source for photosensitization. The following formulae were employed to calculate the time of exposure [29]:

energy fluency = power density × time(3)

power density = power output (mW)/area (cm^2^) (4)

Power density was calculated from power output (132 mW) of the light source and the light-irradiated surface. In this study, experiments on aPDT were performed in a 96-well U-bottom microtiter plate. The sample surface acquired the shape of a cone, with a cross-sectional area of πr^2^, (r, radius = 0.3 cm). Hence, power density was equal to 467.1 mW/cm^2^ (derived from Equation (4)). Energy fluency for the diode laser was set at 84.1 J/cm^2^ and used in aPDT studies [19]. The time of irradiation was calculated using Equation (3).

### 2.11. Minimum Inhibitory Concentration (MIC)

The MIC of MB, AgNPs, and MB-AgNPs for the test bacteria were determined by the broth microdilution method. Two-fold serial dilution was performed for free MB, AgNPs, and MB-AgNPs with an initial concentration of 1 mg/mL. Overnight bacterial cultures were diluted using PBS, and McFarland standard of 0.5 was maintained by adjusting the optical density (OD). The treated bacterial culture plates were incubated (at 37 °C for 24 h) after light exposure (3 min). After incubation, the MIC of each sample was recorded [30].

### 2.12. Antimicrobial Photodynamic Inactivation of Bacterial Cells

Antimicrobial photodynamic killing of *S. aureus* and *P. aeruginosa* by free MB, AgNPs, and MB-AgNPs was determined [31]. A bacterial suspension of 0.5 McFarland standard was incubated in the dark for 2 h with MIC concentrations of free MB, AgNP, and MB-AgNPs separately. Cell suspension was centrifuged for 5 min at 10,000 rpm after 2 h. The pellets were rinsed gently with sterile PBS and suspended in sterile PBS. The cell suspensions were irradiated with a diode laser for 3 min after treatment with MB, AgNPs, and MB-AgNPs separately. A control experiment was maintained by performing similar experiments in the absence of light. Samples exposed to light (L+) and incubated only in dark (L−) were subjected to serial dilution in PBS. All samples were seeded onto agar plates and allowed to grow at 37 °C for 24 h. The total number of colonies grown on next day of incubation was recorded to determine the log reduction.

### 2.13. Detection of ROS

aPDT in bacteria resulted in the production of ROS. Production of ROS was determined using the substrate 1,3-diphenylisobenzofuran (DPBF). A stock of 500 μM DPBF was prepared by dissolving dimethylformamide (DMF) in H_2_O at a ratio of 9:1. The MIC concentration of free MB, AgNPs, and MB-AgNPs was added to bacterial cultures with a McFarland standard of 0.5 and incubated in the dark for 2 h. Cell suspension was centrifuged for 5 min at 10,000 rpm after 2 h. Pellets were washed three times in sterile PBS and again suspended in it. After incubation, all test samples were dispensed in microtiter plate wells, and DPBF was mixed with each sample at a final concentration of 50 μM. Similar experiments were performed under dark conditions as the control. DPBF alone was set for both irradiated and non-irradiated conditions. After irradiation, optical density of the reaction mixture was recorded at 415 nm using a spectrophotometer [32].

### 2.14. Protein Leakage

The leakage of proteins from the bacterial cells was studied after each treatment with free MB, AgNPs, and MB-AgNPs [33]. Overnight bacterial cultures were centrifuged, and the number of bacteria was adjusted to 0.5 McFarland using sterile PBS. The culture suspensions were treated with MIC concentration of free MB, AgNPs, and MB-AgNPs. Bacteria treated with and without MB, AgNPs, and MB-AgNPs were incubated in dark conditions at 37 °C for two hours. The samples were illuminated for 3 min under sterile conditions. Samples were centrifuged for 20 min at 10,000 rpm. Proteins present in the supernatant were estimated using Lowry’s method [34]. CTAB (*N*-cetyl-*N*,*N*,*N*-trimethylammonium bromide) treated cultures were maintained as a positive control.

### 2.15. Biofilm Inhibition Assay

Inhibition of *S. aureus* and *P. aeruginosa* PAO1 biofilm development after aPDT was measured using the crystal violet assay. The test bacteria (0.5 McFarland standard) were added to brain-heart infusion (BHI) broth and incubated in the dark with free MB, AgNPs, and MB-AgNPs for 2 h. The experimental setup containing the samples was irradiated and then allowed to grow for 48 h at 37 °C. Bacteria that adhered to the multitier plates were removed and stained after washing with sterile PBS. The attached bacteria were stained using crystal violet (200 μL of 0.1%) at room temperature for fifteen minutes and washed with sterile PBS. The dye absorbed by biofilms was extracted using ethanol (95%, 100 μL). A similar experimental setup was also maintained in dark conditions. Biofilm formation was quantified spectrophotometrically at 595 nm [19]. The percentage of biofilm inhibition was calculated as follows:

(5) $$\mathrm{Biofilm}\;\mathrm{inhibition}\;\left(\%\right)=\frac{{\mathrm{Control}\;\mathrm{OD}}_{595}-{\mathrm{Test}\;\mathrm{OD}}_{595}}{{\;\mathrm{Control}\;\mathrm{OD}}_{595}}\times 100.$$

### 2.16. Confocal Laser Scanning Microscopy (CLSM)

CLSM analysis was performed to study the outcome of aPDT on the mature biofilm of *S. aureus* and *P. aeruginosa*. Biofilms were developed on glass cover slips (12 mm) in 12-well cell culture plates containing sterile LB broth. Biofilms were permitted to grow on cover slips at 37 °C for 48 days. After incubation, biofilms developed on cover slips were treated with MIC of free MB, AgNPs, and MB-AgNPs and irradiated with a laser. A similar experiment was performed in the dark (without irradiation) which served as the control. The cover slips were washed to remove unadhered cells using sterile PBS after exposure to light. Cover slips with attached biofilms were stained with two dyes, ethidium bromide, and acridine orange. The biofilms after treatment were visualized using CLSM [14].

### 2.17. Statistical Analysis

The results were represented as mean ± standard deviation of triplicate experiments. One-way analysis of variance (ANOVA) was applied to compare multiple means. Statistically significant results were considered only when *p*-values ≤ 0.05.

## 3. Results

### 3.1. Isolation and Identification of Bacteria

**Figure 1:**
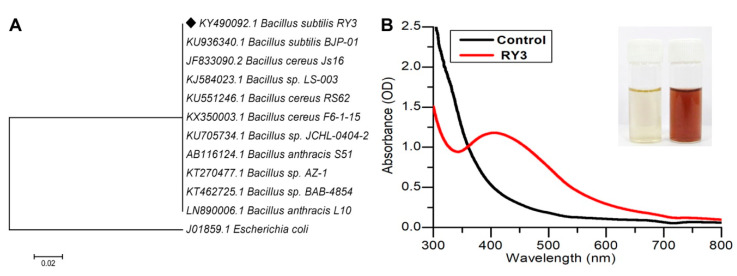
(**A**) Phylogenetic tree of the 16S rDNA sequence of isolate *Bacillus subtilis*. The bar represents 0.02 substitution per nucleotide position. (**B**) UV-Visible absorption spectrum of biogenic silver nanoparticles (AgNPs) using *B. subtilis* RY3 supernatant.

A total of 10 morphologically distinct colonies were isolated from sewage water samples and screened for their ability to synthesize AgNPs. *B. subtilis* RY3 strain was chosen for the biological synthesis of AgNPs from all the bacterial isolates. Molecular identification of RY3 bacterial isolate showed 99% identity to various *Bacillus* sp., primarily with *B. subtilis*. The 16S rDNA sequence of RY3 was deposited in GenBank (NCBI) under the name *B. subtilis* RY3 with an accession number KY490092 (Figure 1A).

### 3.2. Synthesis and Characterization of AgNPs

A change in the color from slight yellow to brick red was observed after 2 h in the reaction mixture when the AgNO_3_ solution was mixed with the culture supernatant and exposed to sunlight. No color change was observed in the controls, i.e., AgNO_3_ solution and culture supernatant. The appearance of a brick red color indicated the formation of AgNPs. A peak at 420 nm in the UV-Vis spectrum further confirmed the formation of AgNPs (Figure 1B).

**Figure 2:**
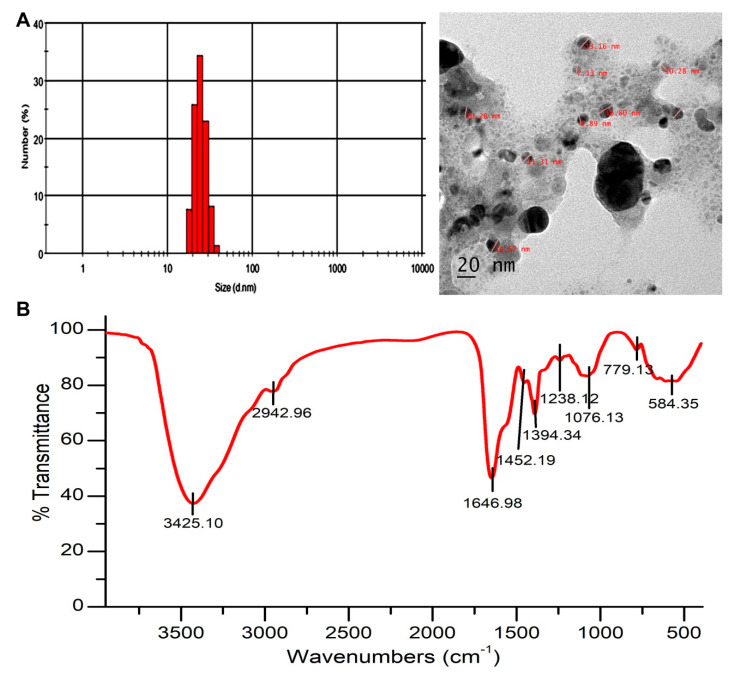
(**A**) Dynamic light scattering (DLS) graph represents the particle size distribution and High-resolution transmission electron microscopy (HR-TEM) micrograph of AgNPs. (**B**) Fourier transform infrared spectroscopic (FTIR) analysis of the biogenic AgNPs.

The DLS histogram of AgNPs revealed that the size was in the range of 20–50 nm, with an average size distribution of 30 ± 5 nm (Figure 2A). Size and shape of the synthesized AgNPs was observed using high resolution HR-TEM (Figure 2A), where spherical nanoparticles with round edges were observed. The particle size of the nanoparticles obtained in DLS was in agreement with High-resolution transmission electron microscopy (HR-TEM) results.

**Figure 3:**
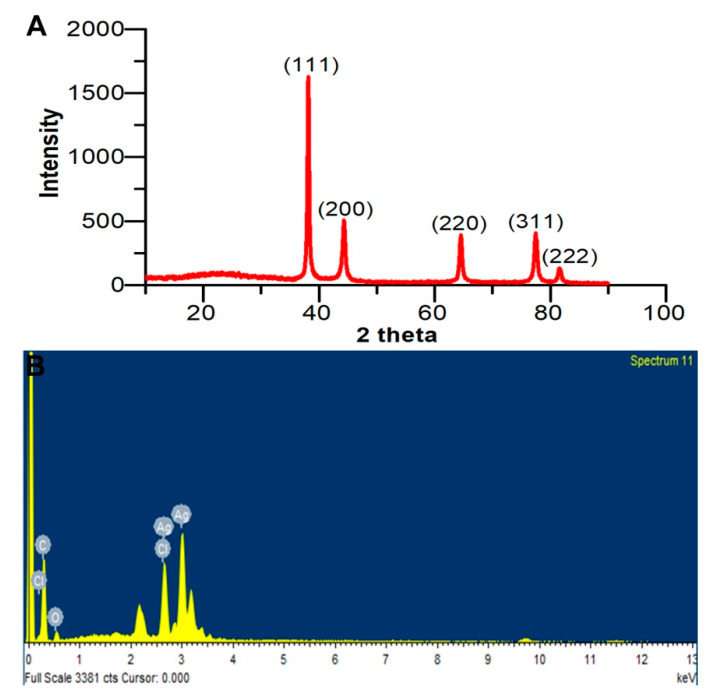
(**A**) X-ray powder diffraction (XRD) profile of the biogenic AgNPs. (**B**) Energy Dispersive X-Ray Analysis (EDX) spectrum of the biosynthesized AgNPs with their corresponding strong Ag signal at 3 keV.

FTIR analysis was performed to examine the possible interaction between Ag and the biomolecules and the functional groups present in the AgNPs. FTIR spectrum (Figure 2B) showed the presence of a band at 1646.98 cm^−1^ and was identified as –NH_2_ stretch vibrations of amide I of protein. A peak observed at 1076.13 cm^−1^ represents –C–O stretch. The wide peak at 3425.10 cm^−1^ represents secondary amides of protein (–N–H). FTIR analysis revealed the presence of carbonyl and amide groups. This may be due to the presence of protein on the surface of AgNPs. The XRD diagram of AgNPs presented four diffraction peaks at 38.12°, 44.24°, 64.5°, and 77.62° corresponding to 111, 200, 220, and 311 planes of Ag, respectively (Figure 3A). The calculated AgNPs lattice parameter correlated with the cubic setting (space group Fm-3m (225)) with unit cell parameter a = b = c = 4.0776Å. The average crystalline size of AgNPs was calculated using the Debye Scherrer equation, where D = 0.94 λ/β cos θ. The average crystalline size for Bragg’s (111) reflection planes was 17 nm.

The EDX spectrum of AgNPs confirmed the presence of Ag. EDX spectrum displayed other elements, such as C and O that confirm organic material as the capping agent on the surface of the AgNPs (Figure 3B).

### 3.3. Preparation and Characterization of MB-AgNPs

**Figure 4:**
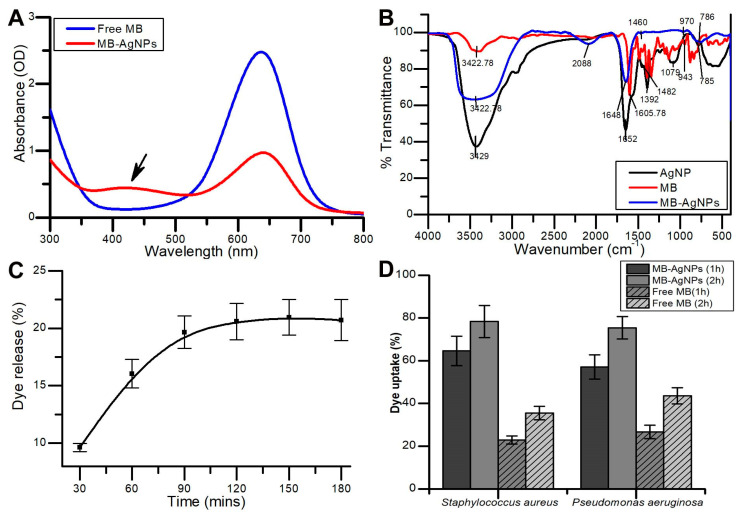
(**A**) UV-Visible absorption spectrum representing the characteristic peaks of AgNPs, methylene blue (MB), and MB-AgNPs. (**B**) Functional groups of AgNPs, MB, and MB-AgNPs are recorded on a FTIR spectrum. (**C**) Release profile of dye from MB-AgNPs studied through in vitro dye release study. (**D**) Bacterial uptake of MB from free MB and MB-AgNPs.

AgNPs were conjugated with MB. The maximum absorption spectrum of AgNPs measured by UV-Vis spectroscopy was at 420 nm due to surface plasmon resonance. A peak at 668 nm for MB-AgNPs indicated the presence of MB. Figure 4A depicts the UV-Vis absorbance spectra of MB-AgNPs. Functional groups of AgNPs, MB, and MB-AgNPs are displayed in Figure 4B. FTIR analysis showed the presence of MB in the MB-AgNPs complex. The spectrum of AgNPs was plotted with bands at 3429, 1652, 1392, 1079, and 785 cm^−1^. The band at 1392 cm^−1^ represented –CH_3_ asymmetric bending vibration of protein. Two bands at 3429 cm^−1^ and 1652 cm^−1^ of AgNPs represents –NH_2_. These groups were thought to be involved in the generation of hydroxyl radicals through photo reaction and were influenced by the surface hydroxyl groups of silver nanoparticles. Among them, the bands at around 1079 and 785 cm^−1^ were recognized as –C–O stretch and –C–H. The stretching and bending vibrations of –N–H group were observed at broad bands of 3429 cm^−1^ and 1652 cm^−1^, respectively. –N–H stretching of secondary amide of the protein and –N–H, C=N and –C–H stretching vibrations of MB were detected at 3422.78, 1605.78, 1482, and 943 cm^−1^, respectively. The characteristic absorption peak of Ag-O-MB was observed at 785 cm^−1^ in both spectrums and it shows that the coupling agent reacted with the hydroxyl group but not with silver. Thus, the composition and structure of AgNO_3_ did not alter the coupling reaction. The sharp and intense groups of MB-AgNPs were identified at 3422.78, 2088, 1648, 970, and 786 cm^−1^ and were associated with the same functional groups as observed for AgNPs and MB.

### 3.4. In Vitro Release and Bacterial Uptake Study

In vitro release of MB from MB-AgNPs was observed. An initial burst release of 16.04% of MB was recorded in the first 1 h. The total MB released after 2 h was 20.48%. Saturation in release of MB from MB-AgNPs was observed after 2 h (Figure 4C). The results of the bacterial uptake study showed that a higher amount of MB was internalized into bacterial cells when treated with MB-AgNPs compared to that with free MB. After 2 h of incubation, 75.4% of MB was internalized in *P. aeruginosa* treated with MB-AgNPs, whereas only 43.6% of MB was internalized in MB-treated cells. In case of *S. aureus*, 78.33% and 35.5% of MB uptake was observed when treated with MB-AgNPs and free MB, respectively (Figure 4D).

### 3.5. Minimum Inhibitory Concentration (MIC)

Antimicrobial activity of MB, AgNPs, and MB-AgNPs against *S. aureus* and *P. aeruginosa* at different concentrations showed dose-dependent antimicrobial activity against the tested organisms. MB-AgNPs exhibited a two-fold higher activity than AgNPs and four-fold higher activity than free MB. MIC value of MB-AgNPs was determined as 125 µg/mL, and all further studies were performed at this concentration.

### 3.6. aPDT on Planktonic Cells

**Figure 5:**
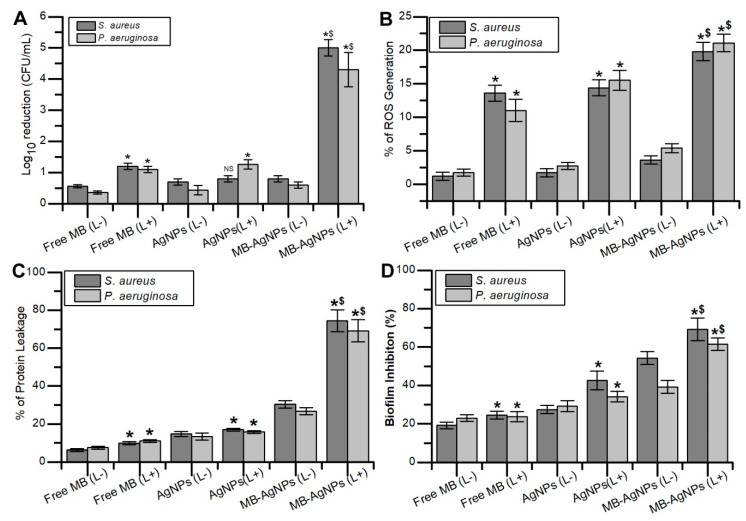
(**A**) Antimicrobial photodynamic killing of free floating bacteria with MB, AgNPs, and MB-AgNPs. (**B**) The percentage of ROS generated after aPDT against *S. aureus* and *P. aeruginosa*. (**C**) The percentage protein leakage from *S. aureus* and *P. aeruginosa* after treatment with MB, AgNPs, and MB-AgNPs. (**D**) Effect of aPDT on biofilm formation in the bacterial samples treated with free MB, AgNPs, and MB-AgNPs in dark and light irradiation conditions. (L+) Light irradiation, (L−) non-irradiation. Standard deviation of three independent experiments are represented on bar diagram with error bars. Asterisk denotes statistical difference compared with the respective dark control (*p* < 0.05). Dollar represents a significant difference compared with free MB with irradiation (*p* < 0.05). NS denotes no statistical significance with respect to control.

The antimicrobial activity of free MB and MB-AgNPs with light irradiation was investigated by the colony count method. MB-AgNPs treatment exhibited a 4.3-log_10_ CFU/mL reduction on *P. aeruginosa* cells after photoactivation, whereas free MB-treated cells showed 1.1-log_10_ reduction. In the case of *S. aureus*, MB-AgNPs showed 5-log_10_ reduction in cells after aPDT, whereas only a 1.2-log_10_ reduction was observed with the free MB (Figure 5A). Increased antimicrobial activity was observed with MB-AgNPs after photoactivation when compared to the free MB.

### 3.7. Detection of ROS

Intracellular ROS produced after photoactivation of MB-AgNPs was measured spectrophotometrically by observing the degradation of DPBF. Photodynamic killing of *S. aureus* and *P. aeruginosa* with free MB and MB-AgNPs was due to the production of ROS. The test results showed 19.81 ± 1.39% of ROS production in MB-AgNPs treated cells of *S. aureus*, whereas only 13.6 ± 1.18% of ROS production was observed with the control. In the case of *P. aeruginosa*, 21.09 ± 1.31% of ROS production was observed in MB-AgNPs treated cells and only 11.03 ± 1.63% of ROS production was observed in the control (Figure 5B).

### 3.8. Protein Leakage

Lowry’s method was employed to estimate the quantity of protein released from bacteria after aPDT. CTAB was used as the control, and the obtained result was considered as 100% protein leakage for reference. Protein leakage in *S. aureus* and *P. aeruginosa* was five-fold higher in MB-AgNPs treated cells compared to that in untreated cells, whereas cells treated with MB showed only two-fold higher protein leakage compared with the control (Figure 5C). Higher protein leakage was observed in MB-AgNPs treated cells after light exposure.

### 3.9. Biofilm Formation

The antibiofilm activity of MB-AgNPs was investigated using the crystal violet assay (Figure 5D). MB-AgNPs treatment resulted in 69.15 ± 5.94% biofilm inhibition in *S. aureus*, whereas MB exhibited 42.55 ± 2.03% inhibition. In *P. aeruginosa*, MB-AgNPs treatment inhibited the biofilm formation by 61.41 ± 3.27% and free dye inhibited the biofilm formation by 34.15 ± 2.65%.

### 3.10. Microscopic Analysis

**Figure 6:**
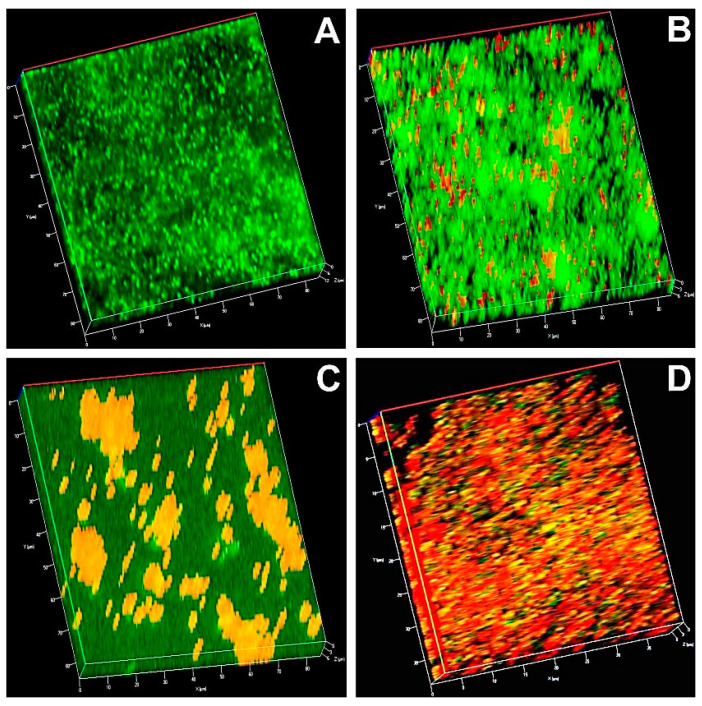
Confocal laser scanning microscopic image of *S. aureus* (**A**) irradiated biofilm (control); (**B**) biofilm treated with MB and irradiated; (**C**) biofilm treated with AgNPs and irradiated; (**D**) biofilm treated with MB-AgNPs and irradiated. Green represents live cells; Red represents dead cells in the biofilm.

**Figure 7:**
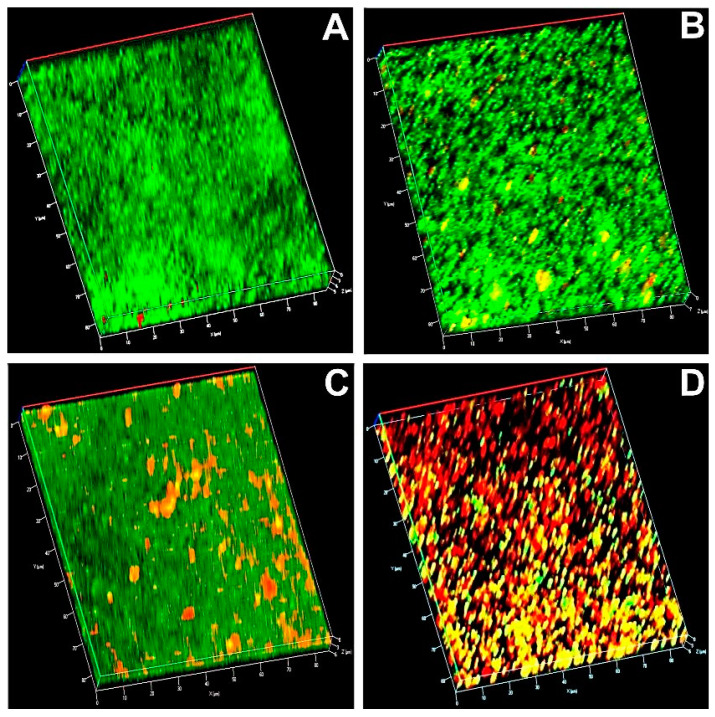
Confocal laser scanning microscopic image of *P. aeruginosa* (**A**) irradiated biofilm (control); (**B**) biofilm treated with MB and irradiated; (**C**) biofilm treated with AgNPs and irradiated; (**D**) biofilm treated with MB-AgNPs and irradiated. Green represents the live cells and red represents dead cells in the biofilm.

In the CLSM image, a dense green fluorescence mat in the control indicated the presence of viable cells (Figure 6 and Figure 7). Biofilms after aPDT with MB and AgNPs showed green and red fluorescence. Predominant red fluorescence in the bacterial samples after aPDT with MB-AgNPs represented more photodestruction of the test bacteria.

## 4. Discussion

Bacterial infections are a major threat to public health worldwide. Conventional antibiotics were recommended earlier for the treatment of bacterial diseases owing to their significant outcome and cost effectiveness. However, in recent years, emergence of multidrug-resistant bacterial strains has been reported [24]. Moreover, biofilm formation poses a major challenge in the treatment of bacterial infections. Bacterial biofilm possesses distinct properties and gene expression patterns compared to their planktonic counterparts [35]. This scenario creates an emergency to design other therapeutic strategies to eliminate multidrug-resistant pathogens and biofilm-mediated infections. aPDT has emerged as a promising alternative compared to conventional methods of bacterial treatment. In recent years, aPDT has emerged as a therapeutic tool against biofilm-mediated infections [36]. In the last two decades, metallic nanoparticles have gained significant attention in medicine and biology as drug delivery vehicles and for diagnostic applications [19]. In recent years, bioengineering gained significant roles in the development of biomaterials that are widely used in different biomedical applications [37]. Metallic NPs have unique physical and chemical properties that facilitate their use as a potential drug or dye carriers [38].

The present study focused on the antibacterial and antibiofilm activities of PS-coated metal nanoparticles against *S. aureus* and *P. aeruginosa*. These are predominant biofilm forming bacteria and are resistant to the traditional antibiotics and are difficult to eliminate from biotic and abiotic surfaces resulting in life threatening situations. Multidrug resistant pathogens such as *S. aureus*, *Corynebacteria*, *Enterococci*, Coagulase negative *Staphylococci*, *Acinetobacter* species, *Pseudomonas* species and *Serratia* are associated with chronic device associated infections. Thus, alternative and effective photodynamic therapy targets multidrug resistant pathogens that cause life threatening infections without allowing the development of any further resistant mechanisms [39]. Although some reports on the effect of PS-coated nanometal conjugates against several bacterial strains are available [14,40], for the first time, the present study demonstrates the extracellular biosynthesis of AgNPs using culture supernatants of bacterial isolate *B. subtilis* RY3 surface coated with MB for the antimicrobial and antibiofilm photodynamic treatment. AgNPs were synthesized using a cell-free supernatant of *B. subtilis* RY3 and silver nitrate as the substrate [41]. AgNP synthesis using *B. subtilis* T-1 isolated from sludge sample has been reported earlier. The synthesis of AgNPs was further confirmed by UV-Vis spectra (Figure 1B) and the results were in correlation with the previous reports [42]. The type of interaction of nanoparticles and dye could be electrostatic interaction, wherein AgNPs can trap MB. UV-Vis spectrum analysis was performed to confirm the conjugation between AgNPs and MB. Crystalline NPs with face-centered cubic phase have been confirmed by XRD analysis [43].

FTIR spectra (Figure 2B) of the AgNPs displayed absorption peaks at 3425.10, 1646.98, and 1076.13 cm^−1^, which correspond to N–H, –NH_2_, and –C–O groups, respectively. The presence of above groups confirmed the existence of a protein coat on the surface of AgNPs. Earlier reports have underlined the fact that biological molecules have significant properties to perform dual function (reducing and capping) in aqueous medium [44]. Furthermore, the study investigated the interactions with AgNPs and the related advantages due to this interaction for antimicrobial activity [45]. Previous reports discussed the antimicrobial activity of AgNPs and its mechanism of action which includes inhibition of DNA synthesis and binding to the proteins on the surface of the microbial cell wall that leads to cell damage [46]. The present result of interaction between MB and proteins coated onto the AgNPs was supported by a previous report where gold NPs were conjugated with MB through electrostatic interaction [19]. Sustained release profile of MB could be assigned to the electrostatic interaction between MB and AgNPs. The sustained release of MB from MB-AgNPs can facilitate recurring treatment using aPDT after single illumination [28]. The presence of negative charge around the bacterial cell wall allowed the PS to bind to the bacterial cell, and the intrinsic properties of AgNPs potentially increased the internalization of the dye into the bacterial cell. The dye uptake result explains the potential of AgNPs as dye delivery agents. Previous reports have also suggested the potential of NPs to deliver a high amount of dye into the bacterial cell when compared to the free dye [18]. In a study, enhanced antimicrobial effect was reported against *P. aeruginosa* and *S. aureus* when polymethylmethacrylate doped with porphyrin and silver nanoparticle were irradiated for 2 h. It is evident from the study that enhanced antibacterial activity is due to the interaction of silver nanoparticles and photosensitizer [5]. Similarly, green synthesized silver nanoparticles and zinc phthalocyanine composites showed improved antimicrobial activity against *E. coli*, *S. aureus* and methicillin resistant *S. aureus* when irradiated with a laser of 660 nm wavelength for 10 min. The nanoconjugates enhanced the production of ROS and improved phototoxicity in test bacteria [15]. The dark toxicity of MB in *P. aeruginosa* and *E. coli* was reduced when encapsulated on mesoporous silica nanoparticles along with excellent photoinactivation ability. Nanoparticles in aPDT together with traditional photosensitizers eliminated the limitations associated with these PS [47]. The results of the present study are comparable with that of previous literature showing an enhanced aPDT of Gram-negative and Gram-positive bacteria rather than that of free dye and nanoparticles.

Incubation of the bacterial suspension with MIC concentration of free MB, AgNPs, and MB-AgNPs in the dark for 2 h did not exhibit significant antibacterial activity. In contrast, light-treated test bacteria in the presence of MIC concentrations of MB, AgNPs, and MB-AgNPs resulted in a significant reduction of viable bacterial count. Likewise, in a previous study, two phenothiazinium dyes such as methylene blue and toluidine blue encapsulated on mesoporous nanoparticles efficiently inactivated *E. coli*, *P. aeruginosa*, and *S. aureus* when irradiated with a red light of wavelength 620 nm even at lower concentration of dyes [48]. It has been already reported that aPDT using a red diode laser as the light source and MB as the PS effectively disturbs biofilms of oral pathogens [36]. It is a well described fact that the degree of photodynamic inactivation is strongly dependent on intracellular ROS production (Figure 5B). It was reported that photosensitizers in the presence of light generates highly cytotoxic reactive species such as hydroxyl free radicals or singlet oxygen. MB is also reported as phototoxic to bacteria and fungi due to their ability to produce ROS [19]. Currently, MB is used in antimicrobial therapy at the clinical level due to their attractive properties such as a high quantum yield of singlet oxygen, low mammalian cell toxicity, and hydrophilicity. MB encapsulated chitosan nanoparticles showed significant inhibition of biofilm of *S. aureus* and *P. aeruginosa* which was more than >2log_10_ CFU reduction [49]. Protein leakage assay results confirmed the photodestruction of bacterial cells when treated with MB-AgNPs and exposed to light. The results of protein leakage after aPDT confirmed damage to the cytoplasmic membrane [50,51]. CLSM micrographs of biofilms subjected to aPDT showed photodestruction of the biofilm treated with MB-AgNPs and exposed to light of appropriate wavelength [52,53]. Furthermore, previous reports have shown complete elimination of pathogenic bacterial biofilm using antimicrobial photodynamic inactivation [54,55]. The greater bacterial killing through PDT may be due to light parameters used (power density of light, energy fluence, time of exposure, incubation time and wavelength of light) and encapsulation of dye on nanoparticles which limits the ability of bacteria to pump out MB. Sustained release and higher bacterial uptake of AgNPs-MB contributed to the greater phototoxicity in test bacteria [56]. The enhanced phototoxicity observed in test bacteria using MB-AgNPs is due to the higher bacterial uptake of MB from nanoparticles followed by the production of higher amounts of ROS. The present study demonstrated that silver nanoparticles surface coated with MB enhanced the aPDT of MB against planktonic cells and biofilms of Gram-negative and Gram-positive bacteria.

## 5. Conclusions

This work synthesized silver nanoparticles through an ecosystem friendly biological route and inspected the antibacterial and antibiofilm action of MB-AgNPs on test bacteria *S. aureus* and *P. aeruginosa*. The AgNPs and nanodye conjugates were characterized using UV-Visible spectroscopy, FTIR, TEM, DLS, XRD, and SEM-EDX. The enhanced antibacterial activity was observed in both bacterial species compared with free MB and AgNPs that contributed a greater amount of ROS production as detected in fluorescence spectroscopy. Further biological assays confirmed that the enhanced antibiofilm activity of MB-AgNPs after light exposure was due to the ability of nano-dye conjugates to reach the deeper layers of biofilm and exhibit greater phototoxicity than free MB. This is the first report on biologically synthesized AgNPs conjugated with MB for aPDT. The use of MB-AgNPs in aPDT can be further explored as an alternative strategy to conventional antibacterial methods.

## References

[1] Mendes F., Paulo A., Santos I. (2011). Metalloprobes for functional monitoring of tumour multidrug resistance by nuclear imaging. Dalton Trans..

[2] Yang Z., Chen B., Pei X., Shangguan F. (2012). Multiplex analysis of tumor multidrug-resistance genes expression with photonic suspension array. Analyst.

[3] Mulani M.S., Kamble E.E., Kumkar S.N., Tawre M.S., Pardesi K.R. (2019). Emerging strategies to combat ESKAPE pathogens in the era of antimicrobial resistance: A review. Front. Microbiol..

[4] Gupta A., Mumtaz S., Li C.-H., Hussain I., Rotello V.M. (2019). Combatting antibiotic-resistant bacteria using nanomaterials. Chem. Soc. Rev..

[5] Lyutakov O., Hejna O., Solovyev A., Kalachyova Y., Svorcik V. (2014). Polymethylmethacrylate doped with porphyrin and silver nanoparticles as light-activated antimicrobial material. RSC Adv..

[6] Maiti S., Krishnan D., Barman G., Ghosh S.K., Laha J.K. (2014). Antimicrobial activities of silver nanoparticles synthesized from *Lycopersicon esculentum* extract. J. Anal. Sci. Technol..

[7] Predoi D., Popa C., Chapon P., Groza A., Iconaru S. (2016). Evaluation of the antimicrobial activity of different antibiotics enhanced with silver-doped hydroxyapatite thin films. Materials.

[8] Prabhu S., Poulose E.K. (2012). Silver nanoparticles: Mechanism of antimicrobial action, synthesis, medical applications, and toxicity effects. Int. Nano Lett..

[9] Naik A.J.T., Ismail S., Kay C., Wilson M., Parkin I.P. (2011). Antimicrobial activity of polyurethane embedded with methylene blue, toluidene blue and gold nanoparticles against *Staphylococcus aureus*; illuminated with white light. Mater. Chem. Phys..

[10] Hanakova A., Bogdanova K., Tomankova K., Pizova K., Malohlava J., Binder S., Bajgar R., Langova K., Kolar M., Mosinger J. (2014). The application of antimicrobial photodynamic therapy on *S. aureus* and *E. coli* using porphyrin photosensitizers bound to cyclodextrin. Microbiol. Res..

[11] Wu J., Xu H., Tang W., Kopelman R., Philbert M.A., Xi C. (2009). Eradication of bacteria in suspension and biofilms using methylene blue-loaded dynamic nanoplatforms. Antimicrob. Agents Chemother..

[12] Khan S., Khan S.N., Meena R., Dar A.M., Pal R., Khan A.U. (2017). Photoinactivation of multidrug resistant bacteria by monomeric methylene blue conjugated gold nanoparticles. J. Photochem. Photobiol. B Biol..

[13] Junqueira M.V., Borghi-Pangoni F.B., Ferreira S.B.S., Rabello B.R., Hioka N., Bruschi M.L. (2016). Functional polymeric systems as delivery vehicles for methylene blue in photodynamic therapy. Langmuir.

[14] Misba L., Kulshrestha S., Khan A.U. (2016). Antibiofilm action of a toluidine blue O-silver nanoparticle conjugate on *Streptococcus mutans*: A mechanism of type I photodynamic therapy. Biofouling.

[15] Chen J., Yang L., Chen J., Liu W., Zhang D., Xu P., Dai T., Shang L., Yang Y., Tang S. (2019). Composite of silver nanoparticles and photosensitizer leads to mutual enhancement of antimicrobial efficacy and promotes wound healing. Chem. Eng. J..

[16] Tawfik A.A., Noaman I., El-Elsayyad H., El-Mashad N., Soliman M. (2016). A study of the treatment of cutaneous fungal infection in animal model using photoactivated composite of methylene blue and gold nanoparticle. Photodiagnosis Photodyn. Ther..

[17] Tunçel A., Öztürk İ., Ince M., Ocakoglu K., Hoşgör-Limoncu M., Yurt F. (2019). Antimicrobial photodynamic therapy against *Staphylococcus aureus* using zinc phthalocyanine and zinc phthalocyanine-integrated TiO_2_ nanoparticles. J. Porphyr. Phthalocyanines.

[18] Sherwani M.A., Tufail S., Khan A.A., Owais M. (2015). Gold nanoparticle-photosensitizer conjugate based photodynamic inactivation of biofilm producing cells: Potential for treatment of *C. albicans* infection in BALB/c mice. PLoS ONE.

[19] Khan S., Alam F., Azam A., Khan A.U. (2012). Gold nanoparticles enhance methylene blue-induced photodynamic therapy: A novel therapeutic approach to inhibit *Candida albicans* biofilm. Int. J. Nanomedicine.

[20] Das V.L., Thomas R., Varghese R.T., Soniya E.V., Mathew J., Radhakrishnan E.K. (2014). Extracellular synthesis of silver nanoparticles by the *Bacillus* strain CS 11 isolated from industrialized area. 3 Biotech.

[21] Dong Z.-Y., Narsing Rao M.P., Xiao M., Wang H.-F., Hozzein W.N., Chen W., Li W.-J. (2017). Antibacterial Activity of Silver nanoparticles against *Staphylococcus warneri* synthesized using endophytic bacteria by photo-irradiation. Front. Microbiol..

[22] Tamura K., Peterson D., Peterson N., Stecher G., Nei M., Kumar S. (2011). MEGA5: Molecular evolutionary genetics analysis using maximum likelihood, evolutionary distance, and maximum parsimony methods. Mol. Biol. Evol..

[23] Shivaji S., Madhu S., Singh S. (2011). Extracellular synthesis of antibacterial silver nanoparticles using psychrophilic bacteria. Process Biochem..

[24] Jain N., Bhargava A., Majumdar S., Tarafdar J.C., Panwar J. (2011). Extracellular biosynthesis and characterization of silver nanoparticles using *Aspergillus flavus* NJP08: A mechanism perspective. Nanoscale.

[25] Raju D., Paneliya N., Mehta U.J. (2014). Extracellular synthesis of silver nanoparticles using living peanut seedling. Appl. Nanosci..

[26] AbdelRahim K., Mahmoud S.Y., Ali A.M., Almaary K.S., Mustafa A.E.-Z.M.A., Husseiny S.M. (2017). Extracellular biosynthesis of silver nanoparticles using *Rhizopus stolonifer*. Saudi J. Biol. Sci..

[27] Deobagkar D., Kulkarni R., Shaiwale N., Deobagkar D. (2015). Synthesis and extracellular accumulation of silver nanoparticles by employing radiation-resistant *Deinococcus radiodurans*, their characterization, and determination of bioactivity. Int. J. Nanomed..

[28] Usacheva M., Layek B., Rahman Nirzhor S.S., Prabha S. (2016). Nanoparticle-mediated photodynamic therapy for mixed biofilms. J. Nanomater..

[29] Rolim J.P.M.L., De-Melo M.A.S., Guedes S.F., Albuquerque-Filho F.B., de Souza J.R., Nogueira N.A.P., Zanin I.C.J., Rodrigues L.K.A. (2012). The antimicrobial activity of photodynamic therapy against *Streptococcus mutans* using different photosensitizers. J. Photochem. Photobiol. B Biol..

[30] Das B., Dash S.K., Mandal D., Ghosh T., Chattopadhyay S., Tripathy S., Das S., Dey S.K., Das D., Roy S. (2015). Green synthesized silver nanoparticles destroy multidrug resistant bacteria via reactive oxygen species mediated membrane damage. Arab. J. Chem..

[31] Ichinose-Tsuno A., Aoki A., Takeuchi Y., Kirikae T., Shimbo T., Lee M.-C., Yoshino F., Maruoka Y., Itoh T., Ishikawa I. (2014). Antimicrobial photodynamic therapy suppresses dental plaque formation in healthy adults: A randomized controlled clinical trial. BMC Oral Health.

[32] Erbas S., Gorgulu A., Kocakusakogullari M., Akkaya E.U. (2009). Non-covalent functionalized SWNTs as delivery agents for novel Bodipy-based potential PDT sensitizers. Chem. Commun..

[33] Thombre R.S., Shinde V., Thaiparambil E., Zende S., Mehta S. (2016). Antimicrobial activity and mechanism of inhibition of silver nanoparticles against extreme halophilic archaea. Front. Microbiol..

[34] Lowry O.H., Rosebrough N.J., Farr A.L., Randall R.J. (1951). Protein measurement with the Folin phenol reagent. J. Biol. Chem..

[35] de Melo W.C., Avci P., de Oliveira M.N., Gupta A., Vecchio D., Sadasivam M., Chandran R., Huang Y.-Y., Yin R., Perussi L.R. (2013). Photodynamic inactivation of biofilm: Taking a lightly colored approach to stubborn infection. Expert Rev. Anti. Infect. Ther..

[36] Cieplik F., Tabenski L., Buchalla W., Maisch T. (2014). Antimicrobial photodynamic therapy for inactivation of biofilms formed by oral key pathogens. Front. Microbiol..

[37] Predoi D., Iconaru S., Predoi M. (2018). Bioceramic layers with antifungal properties. Coatings.

[38] Mura S., Couvreur P. (2012). Nanotheranostics for personalized medicine. Adv. Drug Deliv. Rev..

[39] Anju V.T., Paramanantham P., Siddhardha B., Lal S.S., Sharan A., Alyousef A.A., Arshad M., Syed A. (2019). Malachite green-conjugated multi-walled carbon nanotubes potentiate antimicrobial photodynamic inactivation of planktonic cells and biofilms of *Pseudomonas aeruginosa* and *Staphylococcus aureus*. Int. J. Nanomed..

[40] Tawfik A.A., Alsharnoubi J., Morsy M. (2015). Photodynamic antibacterial enhanced effect of methylene blue-gold nanoparticles conjugate on *Staphylococcal aureus* isolated from impetigo lesions in vitro study. Photodiagnosis Photodyn. Ther..

[41] Płaza G.A., Chojniak J., Mendrek B., Trzebicka B., Kvitek L., Panacek A., Prucek R., Zboril R., Paraszkiewicz K., Bernat P. (2016). Synthesis of silver nanoparticles by *Bacillus subtilis* T-1 growing on agro-industrial wastes and producing biosurfactant. IET Nanobiotechnol..

[42] Sharma G., Jasuja N.D., Kumar M., Ali M.I. (2015). Biological synthesis of silver nanoparticles by cell-free extract of *Spirulina platensis*. J. Nanotechnol..

[43] Fouad H., Li H.J., Ding Y.M., Yu B.T., El-Shakh A., Ghulam A., Mo J.C. (2017). Synthesis and characterization of silver nanoparticles using *Bacillus amyloliquefaciens* and *Bacillus subtilis* to control filarial vector Culex pipiens pallens and its antimicrobial activity. Artif. Cells Nanomedici. Biotechnol..

[44] Sunkar S., Nachiyar C.V. (2012). Biogenesis of antibacterial silver nanoparticles using the endophytic bacterium *Bacillus cereus* isolated from *Garcinia xanthochymus*. Asian Pac. J. Trop. Biomed..

[45] Rudakiya D.M., Pawar K. (2017). Bactericidal potential of silver nanoparticles synthesized using cell-free extract of *Comamonas acidovorans*: In vitro and in silico approaches. 3 Biotech.

[46] Iconaru S.L., Chapon P., Le Coustumer P., Predoi D. (2014). Antimicrobial activity of thin solid films of silver doped hydroxyapatite prepared by sol-gel method. Sci. World J..

[47] Planas O., Bresolí-Obach R., Nos J., Gallavardin T., Ruiz-González R., Agut M., Nonell S. (2015). Synthesis, photophysical characterization, and photoinduced antibacterial activity of methylene blue-loaded amino- and mannose-targeted mesoporous silica nanoparticles. Molecules.

[48] Amin A., Kaduskar D.V. (2019). Comparative study on photodynamic activation of ortho-toluidine blue and methylene blue loaded mesoporous silica nanoparticles against resistant microorganisms. Recent Pat. Drug Deliv. Formul..

[49] Darabpour E., Kashef N., Mashayekhan S. (2016). Chitosan nanoparticles enhance the efficiency of methylene blue-mediated antimicrobial photodynamic inactivation of bacterial biofilms: An in vitro study. Photodiagnosis Photodyn. Ther..

[50] Grinholc M., Nakonieczna J., Fila G., Taraszkiewicz A., Kawiak A., Szewczyk G., Sarna T., Lilge L., Bielawski K.P. (2015). Antimicrobial photodynamic therapy with fulleropyrrolidine: Photoinactivation mechanism of *Staphylococcus aureus*, in vitro and in vivo studies. Appl. Microbiol. Biotechnol..

[51] George S., Kishen A. (2008). Influence of photosensitizer solvent on the mechanisms of photoactivated killing of *Enterococcus faecalis*. Photochem. Photobiol..

[52] Misba L., Zaidi S., Khan A.U. (2017). A comparison of antibacterial and antibiofilm efficacy of phenothiazinium dyes between Gram positive and Gram negative bacterial biofilm. Photodiagnosis Photodyn. Ther..

[53] Paramanantham P., Siddhardha B., Sruthil Lal S.B., Sharan A., Alyousef A.A., Al Dosary M.S., Arshad M., Syed A. (2019). Antimicrobial photodynamic therapy on *Staphylococcus aureus* and *Escherichia coli* using malachite green encapsulated mesoporous silica nanoparticles: An in vitro study. PeerJ.

[54] Mang T., Rogers S., Keinan D., Honma K., Baier R. (2016). Antimicrobial photodynamic therapy (aPDT) induction of biofilm matrix architectural and bioadhesive modifications. Photodiagnosis Photodyn. Ther..

[55] Parasuraman P., Antony A.P., Sruthil Lal S.B., Sharan A., Siddhardha B., Kasinathan K., Bahkali N.A., Dawoud T.M.S., Syed A. (2019). Antimicrobial photodynamic activity of toluidine blue encapsulated in mesoporous silica nanoparticles against *Pseudomonas aeruginosa* and *Staphylococcus aureus*. Biofouling.

[56] Klepac-Ceraj V., Patel N., Song X., Holewa C., Patel C., Kent R., Amiji M.M., Soukos N.S. (2011). Photodynamic effects of methylene blue-loaded polymeric nanoparticles on dental plaque bacteria. Lasers Surg. Med..

